# Supplementary material for: Tumorigenicity decrease in Bcl-xL deficient MDCK cells ensuring the safety for influenza vaccine production
Source: PLoS One. 2024 Dec 16;19(12):e0311069. doi: 10.1371/journal.pone.0311069 (PMC11649150; doi:10.1371/journal.pone.0311069)
Supplement: S1 File — (ZIP) [file pone.0311069.s002.zip › S2_File/3_KEGG_enrichment/scatter/B_vs_A.up_KEGG_enrichment_scatter.pdf]

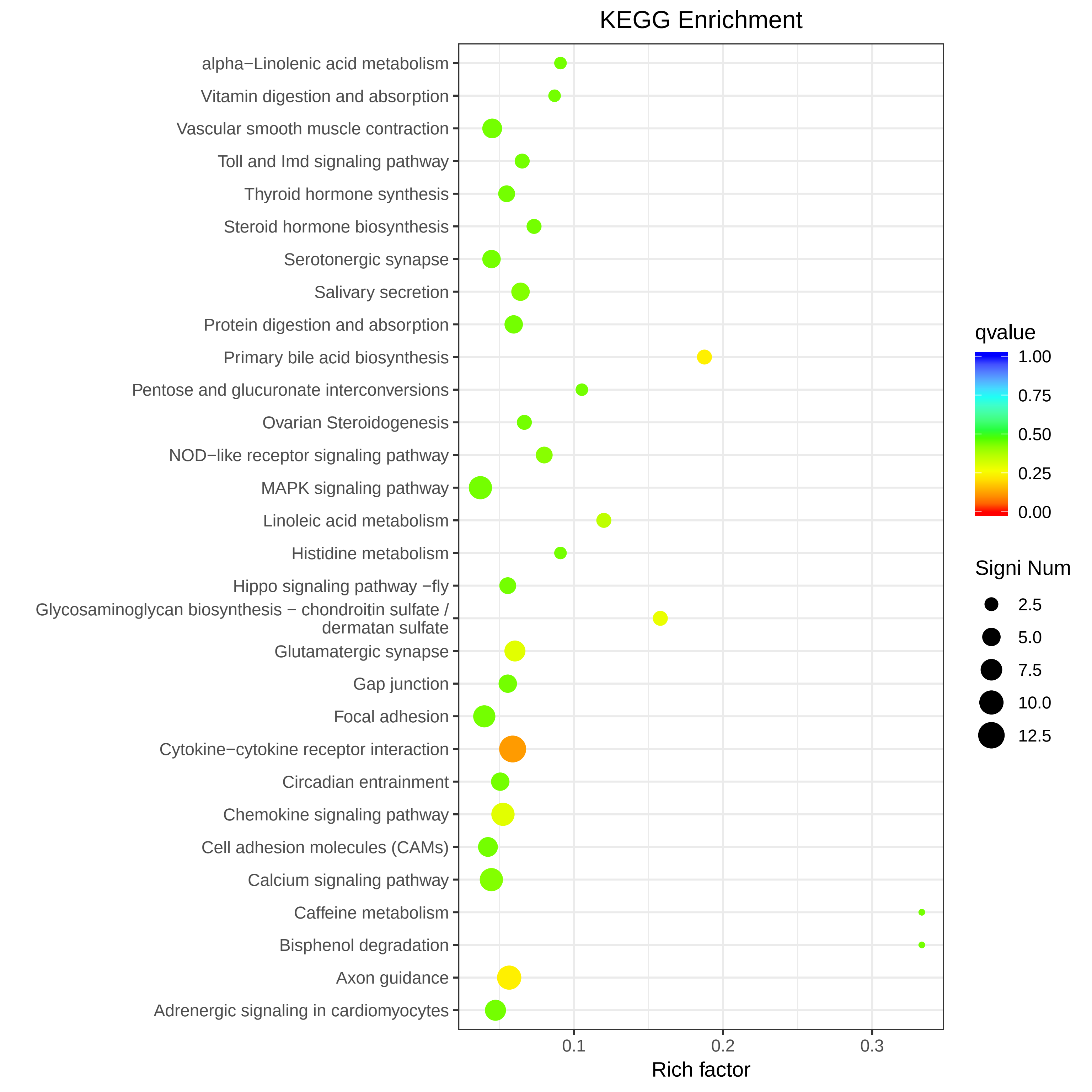

KEGG Enrichment
alpha−Linolenic acid metabolism
Vitamin digestion and absorption
Vascular smooth muscle contraction
Toll and Imd signaling pathway
Thyroid hormone synthesis
Steroid hormone biosynthesis
Serotonergic synapse
Salivary secretion
Protein digestion and absorption
Primary bile acid biosynthesis
Pentose and glucuronate interconversions
Ovarian Steroidogenesis
NOD−like receptor signaling pathway
MAPK signaling pathway
Linoleic acid metabolism
Histidine metabolism
Hippo signaling pathway −fly
Glycosaminoglycan biosynthesis − chondroitin sulfate / dermatan sulfate
Glutamatergic synapse
Gap junction
Focal adhesion
Cytokine−cytokine receptor interaction
Circadian entrainment
Chemokine signaling pathway
Cell adhesion molecules (CAMs)
Calcium signaling pathway
Caffeine metabolism
Bisphenol degradation
Axon guidance
Adrenergic signaling in cardiomyocytes
0.1
0.2
0.3
Rich factor
qvalue
1.00
0.75
0.50
0.25
0.00
Signi Num
2.5
5.0
7.5
10.0
12.5
